# Transient Left Ventricular Dysfunction from Cardiomyopathies to Myocardial Viability: When and Why Cardiac Function Recovers

**DOI:** 10.3390/biomedicines12051051

**Published:** 2024-05-09

**Authors:** Giancarlo Trimarchi, Lucio Teresi, Roberto Licordari, Alessandro Pingitore, Fausto Pizzino, Patrizia Grimaldi, Danila Calabrò, Paolo Liotta, Antonio Micari, Cesare de Gregorio, Gianluca Di Bella

**Affiliations:** 1 Department of Clinical and Experimental Medicine, Cardiology Unit, University of Messina, 98100 Messina, Italy; lucioteresi@gmail.com (L.T.); patgrima@alice.it (P.G.); danilacalabro@gmail.com (D.C.); paololiotta94@gmail.com (P.L.); cesare.degregorio@unime.it (C.d.G.); gianluca.dibella@unime.it (G.D.B.); 2 Department of Biomedical and Dental Sciences and Morphological and Functional Imaging, University of Messina, 98100 Messina, Italy; roberto.licordari@unime.it (R.L.); micariantonio@gmail.com (A.M.); 3 Istituto di Fisiologia Clinica, Clinical Physiology Institute, CNR, 56124 Pisa, Italy; pingi@ifc.cnr.it; 4 Cardiology Unit, Heart Centre, Fondazione Gabriele Monasterio—Regione Toscana, 54100 Massa, Italy; fpizzino@ftgm.it

**Keywords:** transient left ventricular dysfunction, cardiomyopathies, MINOCA, Tako-Tsubo Syndrome (TTS), viability, left ventricular function recovery

## Abstract

Transient left ventricular dysfunction (TLVD), a temporary condition marked by reversible impairment of ventricular function, remains an underdiagnosed yet significant contributor to morbidity and mortality in clinical practice. Unlike the well-explored atherosclerotic disease of the epicardial coronary arteries, the diverse etiologies of TLVD require greater attention for proper diagnosis and management. The spectrum of disorders associated with TLVD includes stress-induced cardiomyopathy, central nervous system injuries, histaminergic syndromes, various inflammatory diseases, pregnancy-related conditions, and genetically determined syndromes. Furthermore, myocardial infarction with non-obstructive coronary arteries (MINOCA) origins such as coronary artery spasm, coronary thromboembolism, and spontaneous coronary artery dissection (SCAD) may also manifest as TLVD, eventually showing recovery. This review highlights the range of ischemic and non-ischemic clinical situations that lead to TLVD, gathering conditions like Tako-Tsubo Syndrome (TTS), Kounis syndrome (KS), Myocarditis, Peripartum Cardiomyopathy (PPCM), and Tachycardia-induced cardiomyopathy (TIC). Differentiation amongst these causes is crucial, as they involve distinct clinical, instrumental, and genetic predictors that bode different outcomes and recovery potential for left ventricular function. The purpose of this review is to improve everyday clinical approaches to treating these diseases by providing an extensive survey of conditions linked with TLVD and the elements impacting prognosis and outcomes.

## 1. Introduction

Recovery of regional or global systolic function is a relatively common piece of evidence observed both spontaneously in some cardiomyopathies and in the setting of optimal medical, interventional, or surgical therapies [1]. Optimal medical therapy (OMT) is associated with increase of left ventricular (LV) function in patients with heart failure (HF) [2]. Particularly, beta-blockers (BBs), mineralocorticoid receptor antagonists (MRAs), sodium-glucose cotransporter-2 inhibitors (SGLT2-is), and either angiotensin-converting enzyme inhibitors (ACEis)/angiotensin receptor blockers (ARBs) or angiotensin receptor neprilysin inhibitors (ARNIs) have been shown to be related to an increase in prognosis and a reduction in hospitalization due to cardiovascular diseases [3]. In the recent DAPA-MODA trial, dapagliflozin administered on top of OMT in stable outpatients with chronic HF determined a global reverse remodeling of cardiac structure, including a reduction in LA volumes and an improvement in LV geometry and NT-proBNP concentrations [4]. Similar results have been observed for empagliflozin in a meta-analysis of randomized controlled trials, suggesting its employment as a potentially promising agent to reverse cardiac remodeling in clinical practice [5].

The pathophysiological mechanism underlying LV dysfunction is multifactorial and includes both abnormal energetics, toxic insults, inflammation, immune responses, excess neurohormonal activation, and excess hemodynamic load. However, patients with a recovered LV ejection fraction (EF) > 50% had a decreased risk of HF hospitalizations, as well as all-cause and cardiovascular mortality compared with HF-preserved ejection fraction (EF) and HF-reduced (r) EF [6].

Although LV dysfunction is usually the consequence of acute and/or chronic heart diseases and its recovery is due to different medical and device therapies (i.e., revascularization and heart valve replacement), in some heart diseases a transient LV dysfunction associate with a spontaneous recovery can be observed when the trigger is eliminated.

**Figure 1:**
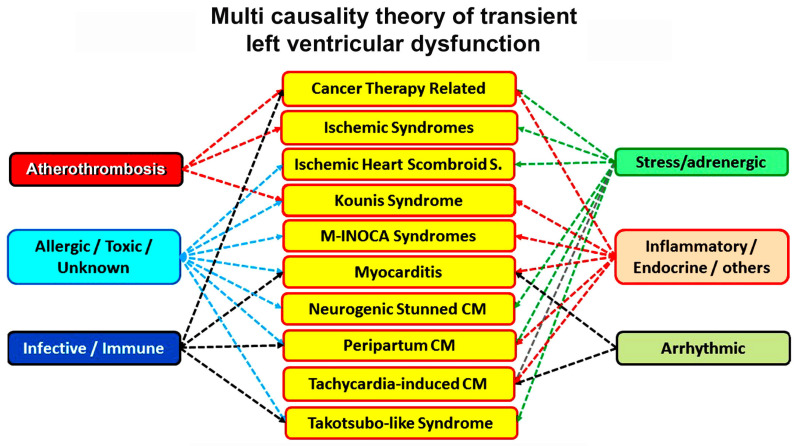
Summary of the main pathogenetic mechanisms of transient left ventricular dysfunction. CM, Cardiomyopathy; S, Syndrome.

Transient left ventricular dysfunction (TLVD) is characterized by the partial or total recovery of ventricular function when the initial triggering factor that determined it is eliminated. TLVD is associated with the absence of significant obstructive epicardial coronary artery disease and its causes often remain underdiagnosed and under-addressed, although they represent an important cause of mortality and morbidity in common clinical practice [7]. From this point of view, this phenomenon may encompass a wide range of disorders including LV dysfunction after emotional or physical stress [8], central nervous system injury [9], histaminergic syndromes [10], inflammatory diseases on an infectious or immune-mediated basis [11], as well as conditions related to pregnancy [12] or other genetically determined syndromes [13] (Figure 1). Also, causes of myocardial infarction with non-obstructive coronary arteries (MINOCA), such as coronary artery spasm, coronary thromboembolism, and spontaneous coronary artery dissection (SCAD), can lead to significant left ventricular dysfunction, with subsequent gradual recovery [14]. TLVD encompasses, therefore, a variety of clinical settings, both ischemic and non-ischemic, that all involve temporary impairment of the left ventricle such as Tako-Tsubo Syndrome (TTS), Kounis syndrome (KS), and other transient ischemic syndromes; Myocarditis; Peripartum Cardiomyopathy (PPCM); and Tachycardia-induced cardiomyopathy (TIC). Timely diagnosis and appropriate management play a critical role in mitigating the significant morbidity and mortality associated with these pathologies and it is imperative to differentiate between each of these, as there may be clinical, instrumental, and genetic factors that can predict the recovery of left ventricular function and consequently affect the prognosis of these patients. Although, it is usually not possible to clearly discern the spontaneous component of the improvement in myocardial function due to OMT, we think that these complex phenomena need to be reviewed according to OMT and the advantage of cardiac imaging.

By implementing an expansive and detailed narrative methodology, this review intends to present the most extensive summary possible of conditions associated with the restoration of systolic function after TLVD. Additionally, it aims to delve into the diverse factors that play a significant role in influencing both the prognosis and the eventual outcomes for patients afflicted with these conditions. Through the meticulous compilation and analysis of relevant studies and findings, this narrative review seeks to provide healthcare professionals with the insights needed to make informed decisions, thereby fostering improved patient care in the clinical setting.

## 2. Tako-Tsubo Syndrome

### 2.1. Pathophysiology

Several pathogenetic mechanisms have been explored in relation to TLVD, particularly in patients with TTS. The typical pattern of regional wall motion abnormalities (RWMAs) in TTS can be explained by the extensive research into β-adrenergic receptors (βARs) [15]. βARs are distributed throughout the LV, with the highest concentration in the LV apex, which is exposed to circulating adrenaline. In contrast, the base of the LV has a greater density of sympathetic nerve terminals and tends to be stimulated via noradrenaline [16,17]. The human myocardium has a higher concentration of β2AR compared to other mammals, with minimal expression of β3AR [18]. Both β1AR and β2AR signal via the canonical stimulatory Gαs pathway, resulting in activation of adenylyl cyclase (AC) and increased intracellular cAMP. At higher agonist concentrations, the β2AR can also signal via the inhibitory Gαi, which physiologically limits the negative effects of Gαs activity [19]. In TTS, excessive release of adrenaline may lead to extreme negative inotropy via the β2AR-Gαi, resulting in apical hypokinesia due to the gradient of adrenergic receptors within the heart [17].

Evidence supporting the involvement of catecholamines in the pathophysiology of TTS comes from studies using Scintigraphic iodine-123–meta-iodobenzylguanidine (mIBG) imaging of the heart [20]. Christensen et al. prospectively evaluated cardiac norepinephrine activity with mIBG scintigraphy and plasma catecholamine levels in 32 patients with TTS and 20 control subjects [21]. The most significant findings showed evidence of myocardial sympathetic hyperactivity and simultaneously increased plasma epinephrine during the subacute phase of TTS, both of which were compared with follow-up in the subacute state. Upon follow-up, there was a complete remission of LV function in the TTS group, and there were no differences in mIBG parameters when compared with the control group [21].

In instances of TTS, there has been evidence of microvascular dysfunction, which could help explain the characteristics of acute ischemic stunning [22]. The sudden surge in catecholamines in TTS likely leads to problems with the endothelium, which could make individuals more susceptible to vasospasms when provoked [23]. The prevalence of vascular dysfunction seems to vary among TTS patients and, in some cases, it has not been found at all [24].While endothelin levels are elevated in TTS and may hypothetically contribute to vasospasms, it is important to note that endothelin levels are also equally high in STEMI patients, where catecholamine-induced contractile dysfunction does not occur [25]. Additionally, catecholamines like adrenaline or dobutamine actually cause vasodilation in coronary arteries, and preclinical models have shown that inducing TTS with catecholamines in the absence of endothelin can lead to apical dysfunction [26] without any abnormality in myocardial perfusion. However, secondary TTS can also occur in patients with mildly-obstructive CAD, who are more likely to have in-hospital complications and recurrences overtime [27]. In addition to the initial contractile dysfunction seen in TTS, it seems that long-term effects of high adrenaline may involve cardiac inflammation. Biopsies from TTS patients have shown mononuclear infiltrates and contraction-band necrosis [28], and cardiac magnetic resonance imaging (CMR) has revealed slowly resolving myocardial swelling. There is also evidence of nitrosative stress in the left ventricular myocardium of TTS patients based on immunohistochemistry studies, and changes in nitric oxide signaling have been demonstrated as well [29,30].

### 2.2. Predictors of Ventricular Recovery and Outcomes

Jurisic et al. explored the time needed for heart function recovery in 406 TTS patients [31]. They found those with delayed recovery of wall motion abnormality were often male patients experiencing physically triggered syndrome, with typical apical ballooning and impaired Left Ventricular Ejection Fraction (LVEF), as well as higher levels of troponin and inflammation markers. Furthermore, factors like male gender, LVEF under 45%, and concurrent acute neurological events predicted a lack of swift recovery within ten days. WMA persisting beyond the initial phase was linked to increased one-year mortality [31].

Meanwhile, Pelliccia et al. in a large case review (54 studies) found that older age, physical stress, and non-typical ballooning patterns were associated with higher long-term mortality in TTS patients [32]. This research further noted that, alongside seeming early recovery, ongoing heart muscle inflammation might progress, potentially resulting in widespread microscopic fibrosis detectable within four months, adversely impacting the long-term outlook for those with TTS [32].

Intense triggers, like physical stress, in susceptible individuals, such as the elderly, could cause more severe myocardial damage. This damage could lead to acute global left ventricular dysfunction (atypical ballooning patterns) and potentially contribute to poorer long-term outcomes in TTS [33].

CMR imaging in TTS [34] typically shows apical ballooning during systole, edema in affected wall areas, and, crucially, the initial absence of Late Gadolinium Enhancement (LGE), which was once considered a distinguishing feature from other heart conditions like myocardial infarction (where LGE is always present) and myocarditis (with LGE in 88% of cases). However, later reports and studies have indicated LGE can occur in TTS [35].

The presence of LGE is clinically significant because it is linked to a worse prognosis in both ischemic and non-ischemic cardiomyopathies [36]. A study involving 20 patients found that LGE was associated with an increased incidence of cardiogenic shock, a longer time for ECG normalization, and delays in the resolution of wall motion abnormalities [37]. These findings emphasize the importance of identifying LGE in TTS patients, as it correlates with more severe clinical outcomes and a longer recovery process.

## 3. Neurogenic Stunned Myocardium

### 3.1. Pathophysiology

Another clinical condition featuring TLVD is neurogenic stunned myocardium, which is a condition where the myocardial wall contractility is impaired due to acute neurological injury [9]. The syndrome has been compared to TTS because of similar echocardiographic findings but higher catecholaminergic distress, usually caused by intracranial bleeding [38]. Despite many comparative studies, conflicting opinions still persist [39].

### 3.2. Predictors of Ventricular Recovery and Outcomes

Neurological severity largely dictates the clinical outcomes in patients with neurogenic stunned myocardium, but myocardial recovery is typically anticipated [40]. After subarachnoid hemorrhage from aneurysm bleeding and treatment, ECG and LV dysfunction represent a matter of clinical concern, due to the challenging management and treatment in cases of simultaneous obstructive CAD.

Accordingly, cardiac dysfunction after SAH seems linked to the damage of discrete sites within the cerebral cortex, hypothalamus, and brainstem, promoting neuroendocrine and autonomic distress causing both cardiac and other organ dysfunction [41]. ECG abnormalities often resolve rapidly, sometimes within a day, and LV function recovers within a few weeks in two-thirds of patients [41,42,43]. Cardiac dysfunction autonomously conveys the SAH patient into a poor outcome setting for high mortality risk, as well as persistence of high troponin T levels after acute cerebral distress [44].

## 4. Kounis Syndrome

### 4.1. Pathophysiology

Kounis syndrome is the term used to describe allergic angina and allergic myocardial infarction. This condition is caused by individual hypersensitivity, characterized by interaction between mast cells, macrophages, T lymphocytes, and inflammatory cytokines [45]. Mast cells can activate macrophages and enhance T cell activation, while macrophage protein 1a and CD169+ macrophages also play a role in this process. T cells are involved in mediating mast cell activation and proliferation, as well as regulating macrophage activity. During hypersensitivity, the mast cells release a variety of inflammatory mediators both locally and into the systemic circulation [46].

These mediators, which include biogenic amines such as histamine, chemokines, enzymes like the neutral proteases chymase and tryptase, and various other substances, play a crucial role in cardiovascular activity. Histamine may lead to coronary vasoconstriction, tissue factor expression, platelet activation, and catecholamine release [47]. Plaque instability can also occur due to the abundant number of macrophages inside the coronary plaques. Additionally, the neutral proteases can activate matrix metalloproteinases, which can degrade collagen and cause plaque erosion and rupture. Tryptase has both thrombotic and fibrinolytic properties. Chymase and cathepsin-D may serve as enzymes to convert angiotensin I into angiotensin II, a significant vasoconstricting substance. Leukotrienes also act as potent vasoconstrictors, with their biosynthesis being heightened during the acute phase of unstable angina [48]. There are three subtypes of Kounis syndrome, but only type 1, coronary vasospasm without thrombosis, has been supposed to cause TLVD [45,46,47,48].

### 4.2. Predictors of Ventricular Recovery and Outcomes

The recovery of left ventricular function in Kounis syndrome is contingent upon the extent of the primary reaction, which is itself influenced by an array of factors such as the patient’s hypersensitivity [49], which may be modified by prior encounters with the allergen. This condition is further complicated by the presence of additional pathologies, including established coronary artery disease [50,51] and mast cell disorders like mastocytosis [52]. Additionally, the location of the immune complex formation, the concentration of the allergen, and the method by which the allergen is introduced into the body (e.g., intravenously as opposed to topically applied) are critical determinants that shape the initial reaction and, thus, the potential for ventricular recovery. In the acute phase, the patients might experience serious complications such as LV dysfunction, pulmonary edema, and cardiac arrhythmias. In type 1 Kounis syndrome, LV functional recovery can be seen in a couple of days after the allergic reaction. Prognosis is generally good in this variant, but the triggering allergens must be avoided [45,46,47,48,49].

## 5. Myocarditis

### 5.1. Pathophysiology

Upon infection, myocarditis triggers the activation of the innate immune response [53]. This activation occurs through the recognition of specific molecular patterns of pathogens and patterns released from damaged cells (DAMPs) via pattern recognition receptors such as Toll-like receptors and nucleotide-binding oligomerization domain-like receptors [54]. The type of pattern recognition receptor and downstream signaling may vary depending on the pathogen or DAMP involved. The activated innate immune cells and cardiac cells then release cytokines, chemokines, interferons, and alarmins, which, in turn, leads to further activation and recruitment of innate immune cells to the heart, including mast cells, neutrophils, dendritic cells, monocytes, and macrophages. In both human and experimental myocarditis, monocytes and macrophages are the main inflammatory cell subsets present [55].

Triggering the innate immune response in the heart can initially protect against viruses, but if this response is overactive or continues too long, it may cause harmful inflammation, leading to tissue damage and heart dysfunction [56]. The resulting pain, anxiety, and alarm signals prompt the bone marrow to produce emergency blood cells, including monocytes. These cells exit the bone marrow, with precursors moving to the spleen to further generate monocytes. This replenishes the spleen’s supply of inflammatory monocytes, which can then travel to the injured heart. This movement of immune cells, especially monocytes, from the spleen to the heart, known as the cardiosplenic axis, is particularly relevant in ischemic heart disease [57]. Understanding and potentially controlling this axis could guide how we direct immune cell migration in inflammatory heart conditions [58].

### 5.2. Predictors of Ventricular Recovery and Outcomes

The trajectory of myocarditis recovery is unpredictable and could result in complete or partial healing or progress to dilated cardiomyopathy without resolution [55]. It may lead to severe systolic dysfunction and ventricular arrhythmias, making prognostic factors critical. Grun et al. found that initial heart failure symptoms can foreshadow long-term outcomes [59]. Severe symptoms, the presence of inflammation, and no beta-blocker usage suggest a greater risk of cardiac mortality or transplant necessity [60]. In contrast, mild symptoms, beta-blocker therapy, and no inflammation are associated with positive outcomes, including high survival rates and reduced need for transplants over five years. Additionally, early ventricular arrhythmias may indicate a higher likelihood of future episodes and studies indicate that the first recurrence of cardiac episodes typically happens within three months following the acute phase of myocarditis [61]. Research by Adegbala et al. revealed that arrhythmias, particularly ventricular tachycardia, are associated with more severe outcomes such as increased hospital mortality and transplant requirements [62]. Factors such as advanced age and existing heart issues heighten the risk of cardiac arrhythmias [62].

In the diagnosis of myocarditis, troponin levels are often elevated initially and decrease rapidly, with the rate of reduction suggesting the resolution of inflammation and a favorable prognosis [63]. Persistent elevation of troponin may imply continue cardiac injury, though the prognostic significance of this is debated; in certain instances, high troponin levels did not correlate with more severe complications [64] nor the duration of giant-cell myocarditis [65]. Contrastingly, levels of NT-proBNP above 4245 pg/mL have been associated with an increased risk of transplantation or cardiac death [66]. Some studies have shown that patients with acute fulminant myocarditis and high troponin but normal BNP levels had poorer outcomes, indicating that this combination of markers might suggest a worse prognosis [67].

Electrocardiographic (ECG) indicators of an unfavorable prognosis include a widened QRS complex and the presence of Q waves, with a wide QRS-T angle (≥100°) being an independent predictor of death and heart failure [68]. A prolonged QTc interval on the ECG also suggests poor clinical outcomes, while ST elevation mimicking a pericarditis pattern is generally associated with better prognosis [69].

Echocardiography plays a vital role in predicting the course of myocarditis. An increase in the LV end diastolic diameter upon admission signifies potential for enduring LV dysfunction and adverse long-term effects [70]. A reduced LVEF of less than 50% seen in the initial echocardiogram signals a higher likelihood of cardiac events [71]. The assessment of baseline LV function, considered a marker for prognosis, when re-evaluated after six months, may provide insight into the longer-term outlook [70]. Additionally, GLS (global longitudinal strain) and strain rates are emerging as potential prognostic tools, with impaired LV strain and strain rates being associated with an elevated risk of negative outcomes [72].

CMR is crucial for predicting heart issues. LGE independently signals higher risk of death from all causes and heart-specific causes: LGE presence often leads to worse LV function and dilatation [73]. Also, the myocarditis involvement of the right ventricle is an independent predictor of worse prognosis [74]. Yet, those without LGE can fare better, despite enlarged LVs and a low ejection fraction, and have a lower rate of sudden cardiac death [73]. Predictive factors include the LGE’s location, size, shape, and spread. Notably, a 10-year study found increased mortality in those with mid-wall LGE in the anteroseptal heart region, versus those without LGE or showing different patterns [75].

## 6. MINOCA

### 6.1. Pathophysiology

Non-atherosclerotic causes of MINOCA are diverse, including coronary artery spasms, microvascular dysfunction, thrombosis or embolism, artery dissection, and mismatches in oxygen supply and demand [76]. Coronary spasms occur due to hypersensitive muscle in the vessels, reacting to internal or external substances like cocaine [77]. Studies indicate that more than 25% of MINOCA patients tested show spasm upon special testing [78]. Microvascular dysfunction is crucial in MINOCA since it accounts for about 70% of coronary resistance, which is hard to detect through angiography as this method primarily reveals larger artery issues [76]. 

In this context, Cardiac Positron Emission Tomography (PET) is recognized as the most effective non-invasive method for detecting microvascular dysfunction [79]. It measures microvascular dysfunction through stress and rest PET scans, quantifying indicators like myocardial blood flow (MBF), myocardial perfusion reserve (MPR), and myocardial flow reserve (MFR), with an MFR below 1.5 indicating decreased flow reserve [80]. Studies by Taqueti et al. revealed that women, even without obstructive coronary artery disease (CAD), often exhibit reduced flow reserve on PET, correlating with a significantly higher risk of cardiovascular diseases [81]. In another research study, Taqueti et al. found that patients without CAD but with abnormal flow reserve were more likely to have diastolic dysfunction and faced a higher risk of being hospitalized for heart failure [82]. 

Single Photon Emission Computed Tomography (SPECT) imaging, while widespread in nuclear cardiovascular diagnostics, faces limitations in MBF due to the low sensitivity and temporal resolution of traditional sodium iodide cameras [83]. The advent of cadmium zinc telluride detectors presents a breakthrough, significantly improving sensitivity and resolution, thus facilitating dynamic SPECT imaging for accurate MBF quantification [84]. While initial studies yield optimistic flow estimates, the advancement of SPECT imaging necessitates broader multicenter trials to enhance its processing techniques and benchmark against conventional methods for flow reserve assessment [85,86].

CMR is emerging as a promising, non-invasive method for assessing myocardial perfusion and flow, thanks to its high spatial resolution, absence of radiation, and strong diagnostic accuracy [87]. It has shown moderate yet significant correlation with PET for identifying microvascular dysfunction (MVD) in women with angina but no obstructive coronary artery disease, as demonstrated by Mygind et al. [88]. Recent research has introduced and validated an automated inline myocardial perfusion mapping technique using CMR, effectively identifying MVD and differentiating it from multivessel epicardial disease [89]. This technique’s validation highlights CMR’s ability to precisely detect MVD, confirming its potential in non-invasively diagnosing coronary microcirculatory disorders.

Likewise, microvascular thrombosis or emboli play roles in MINOCA, with blockages in smaller arterial branches often being culprits. MINOCA often results from thrombosis due to inheritable or lifestyle-related disorders, while embolisms from systemic issues can block small coronary vessels [90].

The MINOCA setting also encloses a rare histaminergic clinical picture typically occurring after eating an improperly prepared/infected fish meal (often tuna fish); it is also known as Scombroid Syndrome [91]. More recently, de Gregorio et al. [92] described the ischemic variant of the syndrome (named as Ischemic Heart Scombroid Syndrome) in some patients referred to the emergency room for typical gastrointestinal syndrome, associated with marked hypotension, chest pain, arrhythmic disorders, and wall motion abnormalities on their echocardiography. These syndromes are caused by the dose-dependent intake of scombrotoxins (histamine, putrescine, and cadaverine) via an improperly prepared (usually raw) fish meal [92,93].

Sometimes, MINOCA is caused by spontaneous coronary dissection, which obstructs the vessel’s lumen and may not show up on angiograms, particularly in the microcirculation [94]. This condition is common in young females and might be linked to hormonal changes during pregnancy, influencing the composition of the vessel wall [94].

### 6.2. Predictors of Ventricular Recovery and Outcomes

Historically regarded as benign due to its low-risk profile and non-obstructive coronary arteries, myocardial infarction with non-obstructive coronary arteries (MINOCA) has been associated with a higher risk of cardiovascular events when compared to individuals without cardiovascular disease [95]. However, the prognosis of MINOCA versus obstructive coronary artery disease (CAD) remains debated, with conflicting results attributed to varying definitions of MINOCA. Some studies suggest a more unfavorable prognosis for myocardial infarction (MI) with obstructive CAD than MINOCA [96,97], whereas others report similar outcomes for both conditions [98,99].

A substantial Japanese retrospective study [100] on 137,678 patients, as for the AHA 2019 classification, reported a total of 13,022 patients with “true MINOCA” and ischemic underpinnings. A “working diagnosis of MINOCA” due to both ischemic and non-ischemic factors was found in another subset of 14,045 patients. This study concluded that both MINOCA groups had a notably increased in-hospital mortality risk equivalent to those with obstructive CAD, suggesting a serious health concern [100]. Key predictors for mortality included factors like age, smoking, diabetes, cancer, chronic lung disease, past stroke, decreased left ventricular function, and elevated creatinine and C-Reactive Protein (CRP) levels [100]. Further emphasizing the gravity of MINOCA, recent research by Bergamaschi et al. on 437 patients highlighted that, with early CMR imaging, the extent of LGE and abnormal T2 mapping are promising predictors of adverse cardiac events over a 3-year follow-up, marking them as significant indicators of high risk [101].

Some patients with Ischemic Heart Scombroid Syndrome may present with severe clinical symptoms (mainly related to the level of the toxin intake) characterized by marked hypotension, shock, respiratory distress, and, at times, requiring advanced medical assistance. However, patients usually experience a full and quick functional recovery [93].

## 7. Peripartum Cardiomyopathy

### 7.1. Pathophysiology

PPCM exemplifies TLVD tied to pregnancy’s hemodynamic shifts [102]. During gestation, increased red blood cells and overall blood volume lead to a 20–30% rise in cardiac output, due to both higher heart rate and 15–25% larger stroke volume. These shifts are notable in the first and second trimesters, often when those with pre-existing heart issues notice symptoms [103]. However, PPCM symptoms typically arise during the peripartum period, suggesting other factors may be involved. Alternative causes like myocarditis are considered, supported by the detection of viral DNA, such as echovirus, Coxsackie, and parvovirus B19, in the cardiac tissue of PPCM sufferers [104].

PPCM might have genetic roots, as familial clustering suggests. Interactions between genetic predispositions and environmental stressors during late pregnancy could heighten PPCM risk [102]. Some research has pinpointed gene mutations in a subset of PPCM cases [105]. Additionally, inflammation may contribute to its onset; elevated concentrations of cytokines, like TNF-alpha and interleukin-6, are seen in PPCM and heart failure patients, indicating a potential inflammatory element in the disease’s progression [106].

### 7.2. Predictors of Ventricular Recovery and Outcomes

PPCM recovery is generally determined by echocardiographic improvements, particularly a rise in LVEF to over 45–55% [107]. Recovery from PPCM, more common than in other heart failure (HF) types, typically happens within 3–6 months of diagnosis. Recovery rates reported vary significantly across studies, spanning 24 to 72% [108]. Several factors influence recovery likelihood and outcomes [12], including initial LVEF [109], African American ethnicity [109,110], CRP levels, hypertensive disorders, and presence of a LV thrombus [109,111]. A meta-analysis by Hosseinpour indicated that patients with higher baseline LVEF, smaller left ventricular diameters, and elevated blood pressure are more likely to recover from PPCM [112].

On the other hand, predictors of poorer outcomes include LV enlargement, presence of LV thrombus [111], right ventricular systolic dysfunction [113], and obesity [114]. African Americans face a particularly high risk of lower recovery rates, extended recovery, worse outcomes, and increased mortality [110]. Patients with coexisting pre-eclampsia have a reduced one-year survival but are more likely to see LV recovery if they survive [115]. Higher biomarker values such as troponin and NT-proBNP have been linked to negative outcomes [116].

CMR studies with smaller cohorts suggest that the presence of LGE indicates an adverse outcome [117,118]. PPCM patients also have notably higher measurements of native T1, extracellular volume (ECV), and T2 when compared to healthy individuals. These CMR parameters correlate with LVEF recovery in PPCM cases; in particular, ECV independently forecasts the recovery of left ventricular function in PPCM patients [119].

## 8. Tachycardia-Induced Cardiomyopathy

### 8.1. Pathophysiology

TIC triggers detrimental changes in heart muscle cell electrophysiology, evident through action potential amplitude reduction, diminished L-type Ca2+ current peak, and extended action potential duration [120]. These alterations contribute to the impaired contraction of the myocytes seen in TIC. Investigations using ventricular myocyte samples from TIC animal models revealed reduced T-tubule and L-type calcium channel density, leading to defective excitation–contraction coupling [121]. TIC is further characterized by hemodynamic shifts, including increased systemic resistance, higher LV filling pressures, and greater LV wall tension. As in other forms of heart failure (HF), TIC elicits an upregulated neurohormonal response to these changes [122]. TIC models showcase a diminished beta-adrenergic response, marked by a drop in beta1-receptors and beta-receptor signaling efficiency. This weakened response undermines the force–frequency relationship crucial for cardiac contractility, potentially exacerbating heart dysfunction under stress. Moreover, TIC may also stem from a depletion of myocardial energy reserves [123].

In animals with TIC, metabolic shifts occur, including decreased creatine, phosphocreatine, adenosine triphosphate, and glycogen [123]. There is also increased activity in the Krebs cycle enzymes and a reduction in the Na-K ATPase pump’s function. These changes suggest mitochondrial damage and dysfunction. Oxidative stress levels rise, leading to cardiomyocyte death and increased susceptibility of mitochondrial DNA to oxidative harm. Subclinical myocardial ischemia might contribute to TIC, indicated by reduced myocardial blood flow and coronary capillary changes. Myocardial hibernation from ischemia could occur, and the heart’s structure might improve if the triggering tachycardia resolves [124].

### 8.2. Predictors of Ventricular Recovery and Outcomes

In a research study focused on a pediatric cohort, it has been established that TIC undergoes resolution in a consistent manner [125]. Predictors of expedited recovery encompass a lower age at presentation, an elevated initial heart rate, the application of mechanical circulatory support, and a higher LVEF. Conversely, the sole precursor of reverse remodeling is a lower baseline size of the LV [125]. Concerning the adult population, Serban et al. have elucidated that patients afflicted with TIC display a sustained dilatation of the LVs, notwithstanding the full restitution of systolic function [126]. These observational results align with the extant literature and underscore the primacy of arrhythmia load reduction in the therapeutic management of TIC-afflicted individuals. The impact of incomplete LV geometric recovery on the exacerbation of LV function upon the reemergence of arrhythmias remains an enigma [126].

A study utilizing CMR imaging has depicted that individuals presenting with persistent atrial arrhythmias who are initially hospitalized for HFrEF exhibit significant enhancement or normalization of LV contractile function within a fortnight of reverting to sinus rhythm in 55.2% of the cases [127]. Those demonstrating TIC resolution—referred to as responders—possessed comparatively reduced chamber measurements and heightened interventricular septum thickness, as evidenced by CMR relative to their non-responder counterparts. Additionally, the incidence of LGE prevailed amongst non-responders; however, its presence was not exclusive to this group. Noteworthily, the pattern of septal mid-wall LGE was exclusively observed in non-responders, suggesting a potential diagnostic marker to differentiate response dynamics within this patient subset [127].

## 9. Left Ventricular Dysfunction in Cancer Treatment

Cancer treatment and survivors face heightened cardiovascular toxicity, making heart disease a major cause of mortality aside from cancer. Lifelong cancer therapies can detrimentally impact heart health. Adverse effects span from left ventricular dysfunction and heart failure to coronary artery disease, heart attacks, high blood pressure, arterial and venous thromboembolism, and arrhythmias [128].

Anthracyclines, chemotherapeutics for solid tumors and blood cancers, are recognized for their heart toxicity. This toxicity can appear as heart failure (HF) soon after treatment or gradually turning into HF. Cardiac dysfunction risk is linked to dose with rates from 5% at 400 mg/m^2^ to 26% at 550 mg/m^2^ [129]. Anthracyclines disrupt DNA by wedging into it and hindering topoisomerase 2, affecting DNA replication and gene expression. They also create reactive oxygen species (ROS) by interacting with iron, damaging cells [130]. Recent studies suggest anthracyclines damage the heart through a mechanism involving cardiac topoisomerase, offering an explanation for the failure of antioxidants in preventing such toxicity [131,132].

After recognizing cardiotoxicity from anthracyclines, various chemo drugs like alkylating agents (cyclophosphamide and ifosfamide) have been found to cause heart muscle disease. These drugs disrupt cell growth, leading to heart issues like abnormal heart rhythms and inflammation, with high doses possibly causing heart inflammation and failure [133,134]. They cause damage to heart cells and endothelium due to toxic by-products [135]. Antimetabolite medications, such as 5-fluorouracil and capecitabine, induce heart toxicity by causing spasms in the coronary arteries and may damage heart cells by promoting oxidative stress and impacting mitochondrial function [130].

Trastuzumab, a humanized antibody targeting HER2—part of the ErbB receptor family overexpressed in certain breast cancer patients—was initially approved for metastatic breast cancer and now also treats HER2-positive breast and gastric cancers [136]. Its incorporation into breast cancer treatment can lead to heart issues in some patients, such as left ventricular dysfunction or heart failure [137]. Studies of trastuzumab’s heart-related side effects showed HER2 is crucial for cardiac health and repairs heart damage [138]. Trastuzumab does not damage the heart directly but interferes with this protective signaling, increasing vulnerability to stressors. While heart function typically recovers within weeks after stopping trastuzumab, some patients experience permanent damage, the reasons for which remain unclear [137].

Vascular endothelial growth factor (VEGF) facilitates the formation of blood vessels, a crucial step for the nourishment of expanding tumors [139]. Anti-VEGF therapies like bevacizumab and TKIs such as sunitinib and sorafenib are linked to serious cardiovascular effects, including high blood pressure, blood clots, and heart damage [140,141]. When used with anthracyclines in breast cancer, bevacizumab can significantly raise the risk of heart failure (HF) [142]. These cardiovascular issues may be influenced by an imbalance in vasoregulation—possibly due to a reduction in vasodilators like nitric oxide and prostacyclin and a spike in the vasoconstrictor endothelin-1, as seen with sunitinib treatment [143,144]. The cardiotoxic potential extends to other TKIs, including dasatinib, nilotinib, and regorafenib. As the list of approved TKIs grows, understanding and mitigating these cardiovascular risks is crucial for the development of safer future therapies [145].

Proteasome inhibitors like bortezomib and carfilzomib, used for multiple myeloma, can cause heart failure—seen in up to 4% and 18% of patients, respectively [146,147]. Their cardiotoxicity is likely due to the disruption of the ubiquitin–proteasome system critical for heart function [148].

Checkpoint inhibitors such as ipilimumab, pembrolizumab, nivolumab, and atezolizumab have a positive impact on the immune response against cancer cells, significantly improving the treatment of melanoma and other malignancies. These inhibitors work by blocking immune regulators, including CTLA-4 (ipilimumab and tremelimumab), PD-1 (nivolumab, cemiplimab, and pembrolizumab), and PD-L1 (atezolizumab, avelumab, and durvalumab), expressed in cancer cells. This inhibition results in a cytotoxic immune response that targets and kills cancer cells. While their exact mechanism is not fully understood, these inhibitors have the potential to activate T cells against non-cancerous tissues, leading to immune-related adverse events [149]. Some of these adverse events can have serious cardiovascular implications such as fulminant myocarditis, myopericarditis, cardiac dysfunction, arrhythmias, or MI, which may ultimately require the discontinuation of the inhibitor [150,151]. A summary of the main conditions related to TLVD is displayed in Table 1.

## 10. Evaluating Myocardial Viability through Magnetic Resonance Imaging

The restore of myocardial segmental function after ischemic reversible and/or irreversible damage is a multifactorial phenomenon observed both in acute and chronic phases of myocardial ischemia. Reversible acute myocardial ischemia due to an increase in oxygen myocardial demand (angina and/or stress test) or occlusion of the coronary artery for less than 20–25 min resulted in abnormalities in LV wall motion without irreversible myocardial damage; it is known as myocardial stunning [153,154].

**Figure 2:**
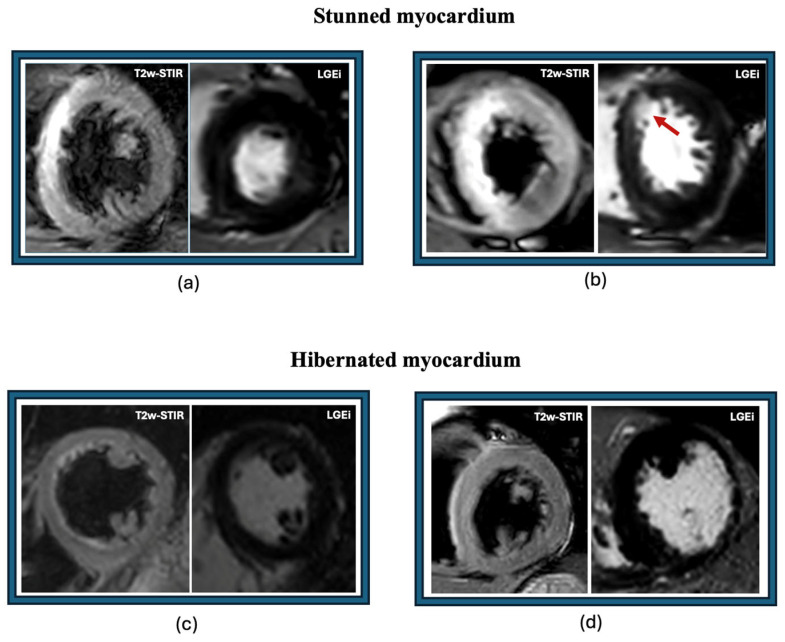
Examples of stunned and hibernated myocardium. (**a**) Aborted AMI treated with PTCA and stent implantation in LAD—WMA 3 in anterior septum and no LGEi. (**b**) Anteroseptal AMI small area of LGEi (red arrow) associated with a diffuse hyperintensity on T2w-STIR images associated with WMA 3. (**c**) Severe LV dysfunction (EF 38%) in absence of LGE and T2w-STIR. (**d**) WMA 3 in anterolateral and inferolateral wall with LGE > 50% and no area of hyperintensity on T2w-STIR. Abbreviations. AMI: acute myocardial infarction; EF: ejection fraction; LAD: left anterior descending artery; LGEi: late gadolinium enhancement imaging; LV: left ventricle; T2w-STIR: T2 weighted short tau inversion recovery; WMA: wall motion abnormality.

In a clinical scenario of an acute myocardial infarction, restoration of systolic function (myocardial stunning) can be observed during the acute phase in a reperfused remote zone contiguous with irreversible scarred damage. These remote peri-infarct zones are characterized by transient wall motion abnormalities (usually 2–3 days) and only myocardial edema without Late Gadolinium Enhancement (LGE). This tissue is also named as salvaged myocardium at CMR. Salvage myocardium is the difference between the area of myocardium at risk (positive T2 weight images) and LGE areas. On the contrary, hibernating myocardium is an ischemic chronic myocardial contractile dysfunction due to reduced coronary blood flow at rest. Hibernating myocardium is a dysfunctional tissue with a high probability of showing a restoration in systolic function. The probability is related to many factors including the presence and the transmural extent of LGE [155]. In the innovative paper by Kim et al., about 80% of dysfunctional segments with no scar at LGE improved contractility after revascularization while segments with < 50% transmural scar extent had 55% improved contractility [156]. In fact, a cut-off of 50% of transmural extent of LGE is one of the CMR criteria of myocardial viability. In patients with previous MI and revascularization there is the probability that segments show normal contractility and <50% transmural scar extent [155]. In a recent study, Di Bella et al. showed that the presence of this tissue (presence of fibrotic segments with contractile activity) had an independent protective effect on the survival of patients with previous MI [157]. Examples are reported in Figure 2.

## 11. Molecular Mechanism of Viable Dysfunctional Myocardium

**Figure 3:**
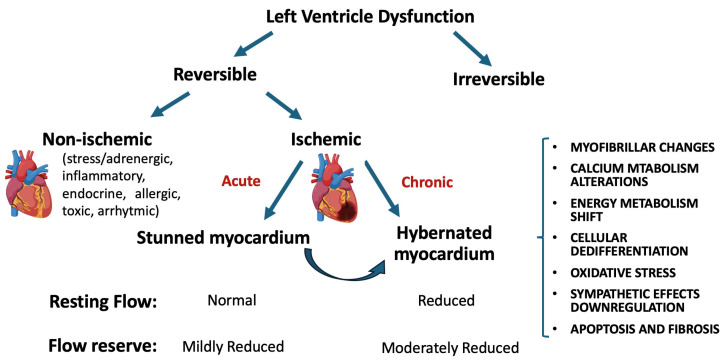
Schematic representation of myocardial responses to left ventricular dysfunction.

In the context of ischemic TLDV, myocardial viability can be inferred when there is an improvement in contractile function following coronary revascularization. This concept encapsulates both ‘stunned’ and ‘hibernating’ myocardium [158]. ‘Stunned myocardium’ refers to a temporary reduction in coronary blood flow (lasting 5–15 min), with normal resting flow, leading to transient LV dysfunction that typically resolves within 24–48 h. In contrast, ‘hibernating myocardium’ is characterized by a sustained impairment due to chronically diminished resting coronary blood flow. However, emerging evidence suggests these conditions may represent a continuum of a singular phenomenon [158]. In a canine study, Shivalkar and colleagues demonstrated that repeated coronary artery obstructions, initially causing transient stunning, eventually lead to persistent dysfunction and subendocardial hypoperfusion [159]. Similarly, Fallovolita et al. observed in pigs that escalating stenosis severity transitions from chronic stunning to hibernating myocardium (Figure 3). This transition correlates with a metabolic shift from lipid to glucose utilization, evident in fluorodeoxyglucose (FDG)-PET imaging. A critical stenosis that depletes the coronary flow reserve appears to initiate this transition, with alterations in coronary flow preceding the development of hibernating myocardium by approximately three months [160].

Various molecular mechanisms have been identified in the development of hibernating myocardium [158,161], intriguingly similarly to those in advanced heart failure, which can be reversed via left ventricular assist device (LVAD) myocardial unloading [162]. These mechanisms include the following:

- Myofibrillar Changes: There is a reduction in thick filament components like troponin, tropomyosin, and myosin light and heavy chains, with an increase in thin filament components such as actin, α-actin, myosin, desmin, and titin [163]. In a mouse model with chronic ischemia caused by vascular endothelium grow factor (VEGF) sequestration, there was a noticeable decrease in the phosphorylation of myosin regulatory light chain 2 (MLC2) and cardiac troponin I (TnI), in line with reduced contractility [164]. The dephosphorylation of MLC2, essential for cardiac muscle contraction, potentially restricts heart contractility in hibernating myocardium. These results indicate that changes in the structure and regulation of myofilament proteins play a role in the heart dysfunction observed in VD myocardium [165].
- Calcium Metabolism Alterations: Impaired calcium regulation via the sarcoplasmic reticulum, due to decreased expression of sarcoendoplasmic reticulum Ca^2+^-ATPase (SERCA), and reduced myofibril sensitivity to calcium contribute to lower electromechanical coupling efficiency. These changes undermine the heart’s ability to contract and relax efficiently, impacting overall cardiac function [160,166,167].
- Energy Metabolism Shift: The suppression of oxygen consumption, alongside an increase in glucose uptake and utilization, marks a metabolic shift in hibernating myocardium. This shift is linked to mitochondrial degeneration, evident through alterations in size and shape, increased glycogen deposition, and decreased expression of proteins crucial for oxidative metabolism and the electron transport chain. These changes signify a move away from efficient ATP production via oxidative phosphorylation, reflecting the cell’s adaptive response to stress and damage [168,169].
- Cellular Dedifferentiation: Expression of embryonic/fetal gene isoforms, including alfa-smooth muscle actin and titin, in adult myocardium. Not only ischemia but also mechanical stretch can induce dedifferentiation, evidenced by the relocation of intercalated disc molecules like β1 integrin, N-cadherin, desmoplakin, and vinculin from the basal to the lateral cell membranes [170,171,172].
- Oxidative Stress: Inducible nitric oxide synthase (iNOS or NOS2) levels, crucial for heart function through nitric oxide (NO) production, were notably increased in human patients with ischemic VD myocardium. NOS2 was also found alongside cyclooxygenase-2 (COX2), responsible for superoxide anion creation. The interaction between superoxide and NO forms peroxynitrite, a potent oxidant, suggesting that the close presence of these enzymes could escalate oxidative stress and harm heart cell structures [173].
- Sympathetic Effects Downregulation: A reduction in α- and β-adrenergic receptors in ischemic cardiomyocytes, despite norepinephrine overflow and reduced presynaptic norepinephrine uptake [174,175].
- Apoptosis and Fibrosis: A key role is played by the downregulation of antiapoptotic and stress proteins like heat shock proteins (HSP)-70, HSP 27, and H11 kinase (H11K) [161]. Dead myocardiocytes are replaced by fibrotic tissue, with associated disruption of cellular junctions like connexin 43 gap junctions, leading to electromechanical dysfunction and creating an arrhythmogenic substrate [176].

## 12. Predictors of Left Ventricular Reverse Remodeling in Dilated Cardiomyopathy

Left ventricular reverse remodeling (LVRR) is strongly related to a good prognosis in dilative cardiomyopathy (DCM), thus LVRR predictors are crucial for prognostic stratification and appropriate disease management. LVRR occurs in approximately 40% of DCM patients in optimal medical therapy two years after diagnosis. However, study comparison can be difficult, because there is not a standardized definition for LVRR, which is usually described as an absolute increase in LVEF ≥ 10% or ≥20% associated with a normalization of LV dimensions or shape [177].

LVRR is likely when adverse remodeling is limited at therapy onset and many predictors have been described. However, these predictors are not accurate and many multiparametric approaches are emerging, but none have been reproduced or externally validated [99,178,179,180]. Female and white people have better LVRR chances. Low systolic blood pressure at diagnosis is related to LVRR, while long QRS, left bundle branch block, or atrial fibrillation at baseline or that occurring during the follow-up are negative prognostic factors [181]. High cystatin C and low HDL-C are associated to poor LVRR. Cistatin C is a marker of renal function and it could be involved in collagen metabolism and heart remodeling, while HDL-C could act as an antioxidant factor [180]. Low BNP and galectin [182] levels at baseline are associated with LVRR [183,184]. Furthermore, two markers of DNA damage, Poly(ADP-ribose) and γ-H2A staining, have been proposed as good LVRR predictors [185]. During a transthoracic echocardiography, low LV size and volume, apical rotation, LV global longitudinal strain > 10%, and an absence of functional mitral regurgitation are associated with LVRR [186,187]. Furthermore, right ventricle (RV) function normalization defined as an RV fractional area change > 35% is a LVRR early predictor and it usually occurs after 6 months of optimal medical therapy [188]. At cardiac magnetic resonance, LVRR is predicted by the absence of LGE, low myocardial T2, and low extracellular volume but not by native T1 at baseline, even if native T1 usually decreases during LVRR [189].

DCM etiology is important. LVRR is more common in cases of tachycardia or hyperthyroidism-induced DCM than in post-partum cardiomyopathy or in myocarditis-induced DCM [190]. On the other hand, a genetic factor is present in at least 20% of patients [191] and a genotype–phenotype correlation is emerging. Nevertheless, genetic assessment using next generation sequencing (NGS) techniques is not a widespread routine and single genetic variants are rare; thus, for statistics, no single mutations are considered but all the pathogenic or probably pathogenic mutations of a gene or even of a group of genes are [185].

Titin (TTN) truncations are the most common mutations in DCM. TTN is a giant protein of the sarcomere with mechanical and signaling functions connecting Z-disc and M-line and its mutations are associated to LVRR [185,192]. Interestingly, in 1005 genotyped DCM patients, Escobar-Lopez et al. [193] found that LVRR occurred more in patients with negative than positive genetics (46.17% vs. 35.33%, *p* < 0.001) with the exception of patients with TTN mutations (46.17% vs. 53.24%). Lamin A/C (LMNA) is an intermediate filament protein that forms the nuclear envelope. It is well known as being mutated in patients with a high risk of malignant arrhythmias and its mutations are also related to poor LVRR [193,194,195].

Other genes’ roles are debated because of controversial findings or poor data. Dal Ferro et al. found that mutations of structural cytoskeleton Z-disc genes such as desmin, dystrophin, and filamin C were related to a low LVRR rate [196], but this was not supported by larger studies [193]. Furthermore, desmosomal (e.g., PKP2, DSC2, and DSG2) and motor sarcomeric genes (e.g., MYH7, ACTC1, and TNNT2) could be related to rare LVRR [196].

## 13. Medical Therapy and Left Ventricular Reverse Remodeling

Previously considered irreversible, cardiac remodeling is now understood to be potentially reversible. Recent decades of research have revealed that the heart can partly restore its structure and function with optimal medical intervention [197]. This process, known as reverse remodeling, occurs in various clinical scenarios and is correlated with better patient outcomes and a more favorable prognosis. As this dynamic process unfolds, it activates mechanisms aimed at reverting the heart’s shape and function towards normalcy. Effective medical treatments have been identified that encourage this reversal, leading to improved heart ventricle shapes, decreased heart size and mass, and better LVEF, ultimately contributing to lower rates of illness and death [197].

Regarding ACEi, in the SOLVD trial with 108 HFrEF patients, those who were asymptomatic showed considerable LV end-diastolic volume (LVEDV) reductions with enalapril treatment at 25 months. Symptomatic patients had reduced LV end-systolic volume (LVESV) and LVEDV, with an LVEF increase at 1 year [198]. The VALIANT and OPTIMAL trials demonstrated ARBs matched ACEis in clinical outcomes for post-myocardial infarction (MI) patients over roughly 25 months and at 6 months, respectively [199,200]. Twenty months after an acute MI, there has been no observed difference between the effects of valsartan and captopril on the improvement of LVEF or the reduction in LVESV and LVEDV [201].

The use of BB therapy has been consistently found to lead to improved survival, clinical outcome, and positive architectural LV changes in the HF population [202,203]. Carvedilol has shown evidence of reducing cardiac remodeling, with improved systolic function and reduced LVESV compared to controls at 6 months post MI, even when significant ACEi use was present in both groups [204].

MRAs are proven to enhance outcomes across the spectrum of HFrEF. The RALES trial connected spironolactone with decreased mortality and cardiac death rates, while EPHESUS showed post-acute myocardial infarction patients with left ventricular dysfunction benefit from eplerenone, with fewer deaths and cardiovascular hospitalizations [205]. Additionally, the EMPHASIS-HF trial demonstrated that eplerenone substantially reduces cardiovascular deaths and heart failure hospitalizations in patients with severe LVEF impairment, also improving overall survival [206]. Treatment with MRAs led to better heart function and size, evidenced by LVEF, LVESV, and LVEDV improvements after 6 to 12 months [207].

The results of the PARADIGM-HF trial showed that ARNI therapy has the potential to reduce hospitalizations for heart failure and improve survival over the course of 27 months when compared to standard HF therapy with ACEi [208]. Additionally, the PROVE-HF study demonstrated that ARNI may also aid in ventricular remodeling in HF patients, as evidenced by significant increases in LVEF and reductions in LVEDV and LVESV indexes at the 12-month mark, even in patients who had already been exposed to ACE inhibitors or ARB [209,210].

There is an increasing amount of evidence that is shedding light on the potential ways in which SGLT2i may work and the effects it has on reversing ventricular remodeling. A recent meta-analysis indicated that empagliflozin not only improved indicators of ventricular remodeling but also indicators of atrial remodeling and overall cardiac function [5]. The analysis found that empagliflozin was more effective than dapagliflozin in reducing LVMi, LVEDV, LVESV, and increasing LVEF. However, further clinical studies are needed to compare the cardiovascular benefits of various SGLT2is. It was also observed that SGLT2is decrease LAVi and E/e’, thereby ameliorating diastolic dysfunction [183].

## 14. Conclusions

TLVD presents a diagnostic challenge with significant clinical implications. This reversible cardiac condition, often escaping early detection, demands enhanced awareness and understanding for optimal clinical outcomes. TLVD encompasses a variety of causes ranging from stress-induced cardiomyopathy to conditions such as myocarditis, Peripartum Cardiomyopathy, and MINOCA, each with unique pathophysiologic underpinnings. This review underscores the importance of differentiating among these etiologies, which necessitate tailored clinical and therapeutic approaches due to their distinct prognostic trajectories. Prompt recognition and accurate diagnosis are paramount in managing TLVD, allowing for the implementation of appropriate treatments that can mitigate morbidity and mortality. The adoption of a nuanced approach to TLVD can lead to improved patient management and better recovery of ventricular function.

## Figures and Tables

**Table 1:** Summary of the main conditions related to transient dysfunction of the left ventricle with references to different pathophysiological mechanisms and the main factors influencing LV recovery.

Causes of TLVD	Pathophysiology Mechanisms	Factors Affecting LV Recovery
Tako-Tsubo Syndrome	Abnormal βARs distribution in the LV with the highest concentration in the apex [16,17]; Excessive release of adrenaline [17]; Extreme negative inotropy via the inhibitory β2AR-Gαi pathway [17].	Male sex, physical triggers, higher Tn levels, higher inflammation markers, LVEF < 45%, and the presence of LGE predict a longer LV recovery process [31,32,33,34,35,36,37].
Kounis Syndrome	Mast cells activate macrophages and enhance T cell activation [46]; Involvement of macrophage protein 1a and CD169+ macrophages [46]; Mast cells release inflammatory mediators into the systemic circulation [46]; Histamine and leukotrienes cause coronary vasoconstriction, platelet activation, and catecholamine release [47].	Immune complexes forming in the heart, higher allergen concentrations, and direct bloodstream injections of allergens lead to more severe cases with pronounced LV dysfunction and extended recovery periods [45,46,47,48,49,50,51,52].
Myocarditis	Recognition of specific molecular patterns of pathogens and DAMPs by Toll-like receptors [55]; Activation of the innate immune response [55]; Release of cytokines, chemokines, interferons, and alarmins [55]; Inflammatory response amplification and recruitment of innate immune cells to the heart, including mast cells, neutrophils, dendritic cells, monocytes, and macrophages [56].	Severe HF symptoms at presentation, presence of inflammation, no beta-blocker usage, ventricular tachycardia, widened QRS complex and the presence of Q waves with a wide QRS-T angle (≥100°), increased LVEDD upon admission, LVEF < 50% at presentation, presence of LGE, and RV involvement are predictors of limited LV recovery [60,61,62,68,69,70,71,72,73,74,75].
MINOCA	Coronary artery spasms [76]; Microvascular dysfunction [76]; Microvascular thrombosis or emboli [90]; SCAD [94].	Higher extent of LGE and abnormal T2 mapping are predictors of a lack of LV recovery and adverse cardiac events [101].
Peripartum Cardiomyopathy	Hypervolemic status with increased red blood cells and overall blood volume lead to a 20–30% rise in cardiac output [103]; Inflammatory state with elevated concentrations of cytokines, like TNF-alpha and interleukin-6, are seen in PPCM [106].	LV enlargement, presence of LV thrombus, RV dysfunction, obesity, African American ethnicity, higher Tn and NT-proBNP values, the presence of LGE, and higher ECV values predict lower LV recovery rates and extended LV recovery [109,110,111,112,113,114,115,116,117,118,119].
Tachycardia-induced cardiomyopathy	Tachycardia causes action potential amplitude reduction, diminished L-type Ca^2+^ current peak, and extended action potential duration [120,121]; Tachycardia induces increased systemic resistance, higher LV filling pressures, and greater LV wall tension [122]; Reduction in the Na-K ATPase pump’s function and mitochondrial damage and dysfunction with elevated oxidative stress levels [123].	Lower age at presentation, an elevated initial heart rate, higher LVEF, and LGE absence are predictors of expedited LV recovery [125,127].
Cancer Treatment cardiotoxicity	Anthracyclines disrupt DNA by wedging into it and hindering cardiac topoisomerase [131,132]; Alkylating agents disrupt cell growth, leading to heart issues like abnormal heart rhythms and inflammation [133,134,135]; 5-FU and capecitabine induce heart toxicity by causing spasms in the coronary arteries [130]; Antibodies that target HER2 disrupt protective signals within cells, making heart muscle cells more susceptible to stress [137]. Anti-VEGF therapies cause imbalance in vasoregulation—possibly due to a reduction in vasodilators like nitric oxide and prostacyclin and a spike in the vasoconstrictor endothelin-1 [143,144]; Proteasome inhibitors disrupt the ubiquitin–proteasome system critical for heart function [146,147,148]; Immune checkpoint inhibitors may activate T cells against non-cancerous tissues, leading to fulminant myocarditis, myopericarditis, cardiac dysfunction, and arrhythmias [150,151].	Anthracyclines: persistent Tn elevation and higher NT-proBNP levels predict severe LV dysfunction and extended LV recovery [152]. Early discontinuation of therapy upon recognition of LV dysfunction and timely initiation of cardioprotective therapy are recognized as approaches capable of promoting LV recovery [152].

Abbreviations: βARs—β-adrenergic receptors, DAMPs—danger-associated molecular patterns, HF—heart failure, LGE—Late Gadolinium Enhancement, LV—left ventricle, LVEDD—Left End Diastolic Diameter, LVEF—Left Ventricle Ejection Fraction, PPCM—Peripartum Cardiomyopathy, RV—right ventricle, SCAD—spontaneous coronary artery dissection, TNF—Tumor Necrosis Factor, 5-FU—5-fluorouracil, Tn—troponin.
